# Comparison of the effectiveness in pain reduction and pulmonary function between a rib splint constructed in the ER and a manufactured rib splint

**DOI:** 10.1097/MD.0000000000010779

**Published:** 2018-05-25

**Authors:** Yoonje Lee, Sang-Hyun Lee, Changsun Kim, Hyuk Joong Choi

**Affiliations:** a Department of Emergency Medicine, Seoul Hospital, Hanyang University, Gyeonggi-do; b Department of Emergency Medicine, Hangang Sacred Heart Hospital, Hallym University, Gangwon-do; c Department of Emergency Medicine, Guri Hospital, Hanyang University, Gyeonggi-do, Korea.

**Keywords:** rib fracture, rib splint, trauma

## Abstract

**Background:**

In the treatment of patients with rib fractures (RFs), pain reduction is the most important consideration. Various studies have examined the effectiveness of treatments administered to RF patients, such as lidocaine patches, IV drugs, nerve blockers, and surgery. In this study, we evaluated the difference in the effectiveness in pain reduction between 2 groups of RF patients: 1 group who received a rib splint constructed in the ER (ER splint) and another group who received a Chrisofix Chest Orthosis (CCO) manufactured rib splint.

**Methods:**

A pilot study for a prospective randomized clinical trial was conducted to compare subjects using the CCO (Group A) with those using the ER splint (Group B) before and after the intervention. The primary outcome was difference in the level of pain based on the visual analogue scale (VAS) and the pulmonary function (PF) variables between before and after intervention in each group during forceful and resting respiration.

**Results:**

A total of 24 subjects were enrolled in this study. The VAS results showed that the intervention was significantly effective in each group (before vs after: Group A resting: 8.50 ± 1.05 vs 4.17 ± 1.33, *P* < .001; Group A forceful: 9.83 ± 0.41 vs 7.17 ± 0.75, *P* < .001; Group B resting: 8.83 ± 1.60 vs 4.50 ± 1.38, *P* < .001; and Group B forceful: 9.67 ± 0.82 vs 7.33 ± 1.51, *P* = .003). The PF variables showed that the intervention was significantly effective in each group (before vs after: Group A, FVC: 2.74 ± 0.92 vs 3.35 ± 0.99, *P* < .001; FEV1: 2.16 ± 0.74 vs 2.57 ± 0.78, *P* = .001; PEF: 235.30 ± 43.06 vs 319.00 ± 51.58, *P* = .004; and Group B, FVC: 2.02 ± 0.49 vs 2.72 ± 0.62, *P* < .001; FEV1: 1.27 ± 0.25 vs 1.91 ± 0.37, *P* < .001; PEF: 216.67 ± 67.49 vs 300.33 ± 87.79, *P* = .003).

**Conclusion:**

Applying either the CCO or the ER splint to RF patients effectively reduced pain, and no significant differences in pain level were observed between these 2 techniques.

## 1 Introduction

In general, rib fractures (RFs) are considered a common injury that results from blunt thoracic trauma.^[1]^ Pleuritic pain and chest wall pain follow RFs. RFs disrupt the stability of the chest wall and decrease the respiratory volume, thus causing restrictive as well as obstructive breathing difficulty, along with unpredictable effects on bronchial secretion. Poor pain control can lead to pulmonary complications as a direct consequence of decreased ability to ventilate.^[2]^ For instance, pneumonia develops in one-third of patients with multiple RFs.^[3]^ Considering that injury to the ribs and the subsequent pain trigger this cascade of negative consequences, optimal pain control is expected to shorten the length of stay and reduce the number of pulmonary complications in RF patients.^[4]^ Thus, in the treatment of patients with RFs, pain reduction is the most important factor.

Numerous methods of pain control have been described for RFs; these methods include systemic opioids or NSAIDs, epidural anesthesia, intercostal nerve blockers, intrathecal opioids, intrapleural analgesia, paravertebral blockers, and transcutaneous electrical nerve stimulation.^[5]^ However, none of these methods has been shown to be completely effective alone, especially in the long term. Additionally, intravascular or oral systemic opioids can cause sedation, decreased respiration, and decreased mental clarity. Each of the other methods is invasive, thus exposing patients to possible complications such as bleeding, pneumothorax, and infection. In addition, these invasive methods are difficult to perform and are impractical in an emergency room (ER) setting.

Malgaigne proposed a thorax-fixing bandage in his book in 1851.^[6]^ Several other adhesive bandaging methods have been advocated. These methods are poorly accepted at present because they decrease the vital capacity of the lung. Recently, rib splints have been designed to simultaneously provide pain reduction and increase vital capacity.^[7]^

Chrisofix Chest Orthosis (CCO) can be used to reduce pain, the length of stay, and the number of pulmonary complications.^[7]^ However, this product is very expensive, and its size is inadequate for application to patients with multiple RFs or a flail chest. Therefore, we have designed a rib splint that is easily constructed using common materials such as adhesive hydrocolloid dressing material, double-sided adhesive tape, and splint, all of which are typically present in the ER.

In this study, we hypothesized that the effects of a rib splint constructed in the ER (ER splint) are similar to those of the CCO rib splint.

## 2 Materials and methods

### 2.1 Study design

This study was a prospective randomized single-blinded human clinical trial. The investigation was approved by the relevant institutional review board, and informed consent was obtained from all subjects. The local ethics committee approved this study (IRB No. 2015-09-013). We registered the study protocol in the Clinical Research Information Service clinical trials database before study initiation (Clinicaltrials.gov NCT03210792).

### 2.2 Study setting and population

**Figure 1:**
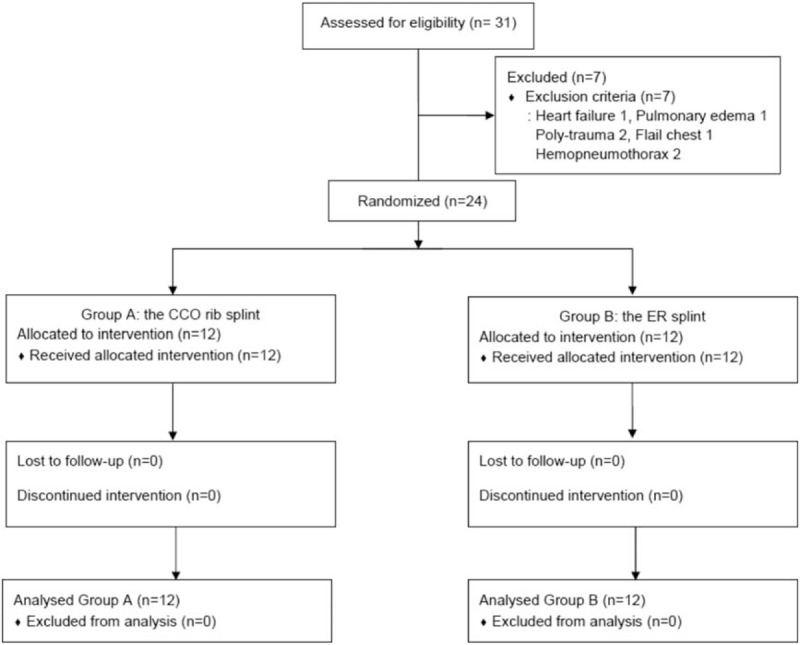
Flow diagram.

This study was conducted at an emergency department of an academic medical center. The primary investigators were emergency physicians and/or emergency residents working in the emergency department. The sample size was calculated based on a pilot study that examined the delta PEF before and after the intervention. The delta PEF (mean ± standard deviation (SD)) values were as follows: Group A delta PEF (81.49 ± 35.97) and Group B delta PEF (83.53 ± 31.59). The sample size was analyzed using G-power 3.1.2 (Heine Heinrish University, Düsseldorf, Germany) with an α error of 0.05 and a power of 0.8. We estimated that 11 participants would be adequate for each group with a 10% dropout rate. Thirty-one patients were initially enrolled. We ultimately enrolled 24 RF patients who were over 18 years of age and who agreed to participate in this study. These patients completed a predesigned written consent form before participating in this study. We excluded 7 patients with cardiopulmonary dysfunction, polytrauma, flail chest, damage to an internal organ, or alcoholism and patients who did not consent to participate (Fig. 1). The patients were divided into the following 2 groups in a randomized order: Group A, which received the CCO rib splint, and Group B, which received the ER splint. The patients were blinded to their group assignments.

### 2.3 Study materials

**Figure 2:**
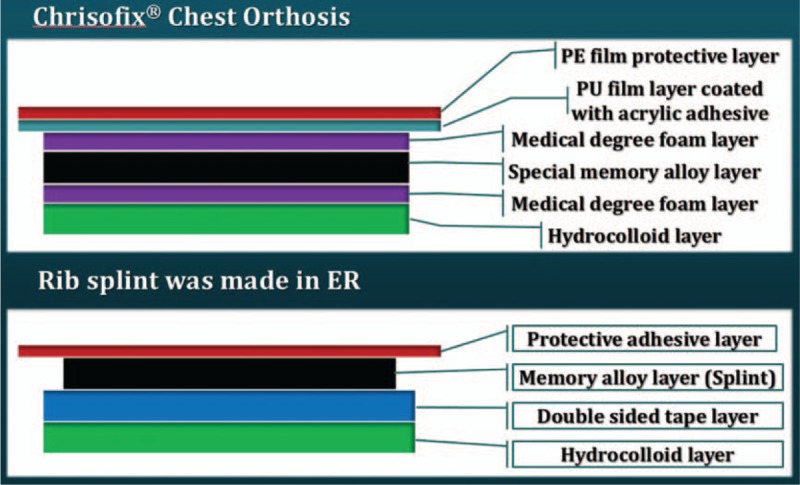
Configurations of Chrisofix Chest Orthosis and the rib splint was made in the ER.

We designed the ER splint in a simplified manner using adhesive hydrocolloid dressing material, double-sided adhesive tape, and a splint in the ER. The ER splint was no different from CCO in terms of its configuration (Fig. 2). We used either the ER splint or the CCO rib splint (Chest Orthosis, Chrisofix AG, Neuhausen am Rheinfall (Switzerland), and ORKRISZ Ltd-s, Budapest, Hungary) in this study.

The following section describes the construction of the ER splint.

**Figure 3:**
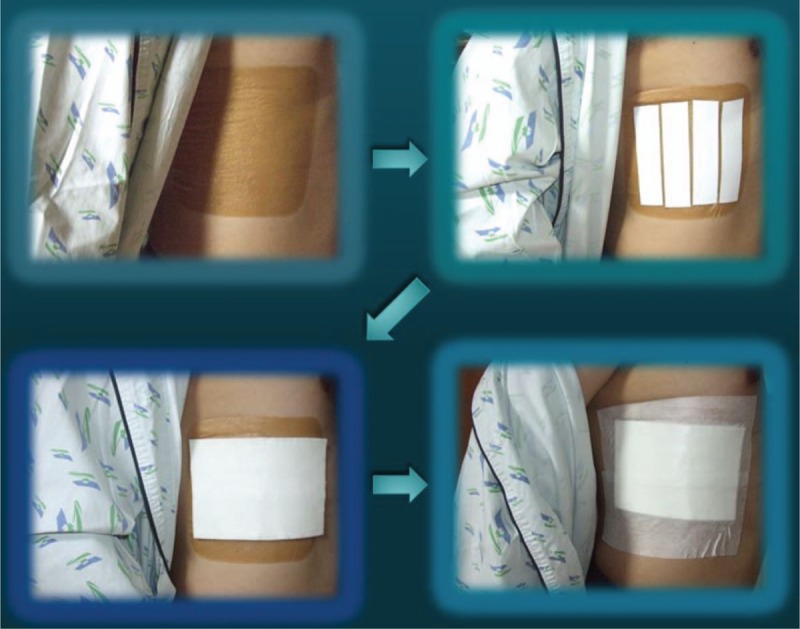
How to make a rib splint in the ER. Marking → Dressing → Attaching the hydrocolloid dressing material → Double-sided tape → Molding a splint → Attaching the splint → Securing with Hypafix Adhesive Bandage.

First, attach adhesive hydrocolloid dressing material to the site of RFs on the patient. Second, attach the double-sided adhesive tape to the hydrocolloid dressing material. Third, attach the splint to the double-sided adhesive tape. Fourth, secure these materials using a Hypafix Adhesive Bandage (Smith & Nephew, Inc., Andover, MA) (Fig. 3).

We used ML3500 MicroLab Spirometer (Woodley Equipment Company Ltd, Bolton, Lancashire, UK) the visual analogue scale (VAS) to assess the level of pain and pulmonary function (PF) in patients with RFs.

### 2.4 Study protocol

First, we performed VAS and spirometer assessments to determine the level of pain in the patients with RFs during resting and forceful respiration. In addition, we applied either the CCO rib splint or the ER splint to the RF patients in a randomized order. At 30 minutes after splint application, we repeated the VAS during resting and forceful respiration and the spirometer assessment. Subsequently, additional pain control was provided via intravascular drug injection.

### 2.5 Outcome measures

We recorded the PF values, including the forced vital capacity (FVC), forced expiratory volume in 1 second (FEV1), peak flow rate (PFR) and the VAS scores during resting and forceful respiration before and after application of the ER splint or the CCO rib splint. We compared the 2 values (before and after intervention) to determine the change in the VAS score and PF in each group during resting and forceful respiration.

### 2.6 Data analysis

The data were compiled using a standard spreadsheet application (Excel, Microsoft, Redmond, WA) and were analyzed using the Statistical Package for the Social Sciences (SPSS) 20.0 KO for Windows (SPSS, Inc., Chicago, IL). We generated descriptive statistics and presented them as frequencies and percentages for categorical data or as the means with the standard deviation (SD) for continuous data.

To evaluate the effects of both the CCO rib splint and the ER splint, the paired *t* test was used to compare the difference in the VAS score and PF after the intervention.

Further, to compare the differences in the VAS score and PF between applying the CCO rib splint and applying the ER splint, an equivalence test was used for continuous variables. Furthermore, to find confounding factors, a multivariable linear regression analysis was performed using several variables (sex, age, number of fractured ribs, fractured side).

For all analyzed data, *P* < .05 was considered statistically significant.

## 3 Results

### 3.1 Baseline characteristics

Twelve patients were enrolled in this study, and none were excluded. The baseline characteristics of the patients are shown in Table 1.

**Table 1:**
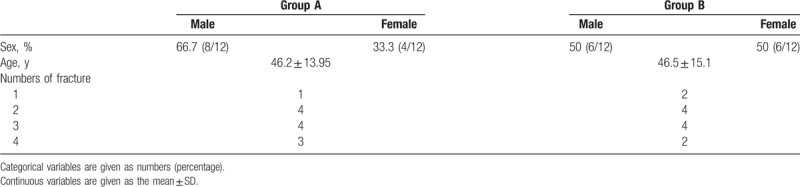
Baseline characteristics.

### 3.2 The effectiveness of the CCO rib splint based on the difference in the VAS scores and pulmonary function variables before and after intervention

Overall, each treatment was significantly effective (Table 2).

**Table 2:**
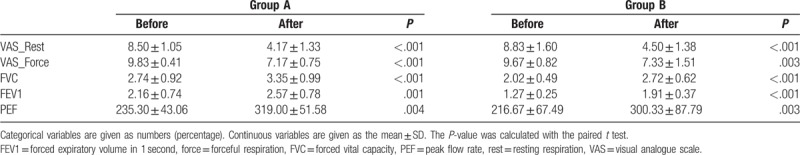
The effectiveness of intervention in each group before and after intervention.

In Group A, application of the CCO rib splint significantly reduced the VAS score during resting respiration (before vs after: 8.50 ± 1.05 vs 4.17 ± 1.33, *P* < .001) and during forceful respiration (before vs after: 9.83 ± 0.41 vs 7.17 ± 0.75, *P* < .001). In addition, the CCO rib splint significantly improved PF (before vs after; FVC: 2.74 ± 0.92 vs 3.35 ± 0.99, *P* < .001; FEV1: 2.16 ± 0.74 vs 2.57 ± 0.78, *P* = .001; PEF: 235.30 ± 43.06 vs 319.00 ± 51.58, *P* = .004).

### 3.3 The effectiveness of the ER splint based on the difference in the VAS scores and pulmonary function variables before and after the intervention

In Group B, application of the ER splint significantly reduced the VAS score during resting respiration (before vs after: 8.83 ± 1.60 vs 4.50 ± 1.38, *P* < .001) and during forceful respiration (before vs after: 9.67 ± 0.82 vs 7.33 ± 1.51, *P* = .003) (Table 2). Furthermore, the ER splint also significantly improved PF (before vs after; FVC: 2.02 ± 0.49 vs 2.72 ± 0.62, *P* < .001; FEV1: 1.27 ± 0.25 vs 1.91 ± 0.37, *P* < .001; PEF: 216.67 ± 67.49 vs 300.33 ± 87.79, *P* = .003).

### 3.4 Comparisons of the equivalence of the effectiveness of the 2 tested interventions

There was no significant difference in pain relief between Groups A and B during resting respiration (%ΔVAS before − %ΔVAS after, Group A vs Group B: 40 ± 15 vs 45 ± 12.5, *P* = .94) or during forceful respiration (%ΔVAS before − %ΔVAS after, Group A vs Group B: 25 ± 12.5 vs 20 ± 15, *P* = .59) (Table 3). There was no significant difference in the improvement in FVC and PEF between Groups A and B (Group A vs B; FVC: 0.61 ± 0.16 vs 0.71 ± 0.19, *P* = .48; PEF: 83.67 ± 39.69 vs 82.93 ± 35.40, *P* = .94). However, there was a statistically significant difference in the improvement in FEV1 between Groups A and B (Group A vs B; FEV1: 0.41 ± 0.13 vs 0.64 ± 0.19, *P* = .03).

**Table 3:**
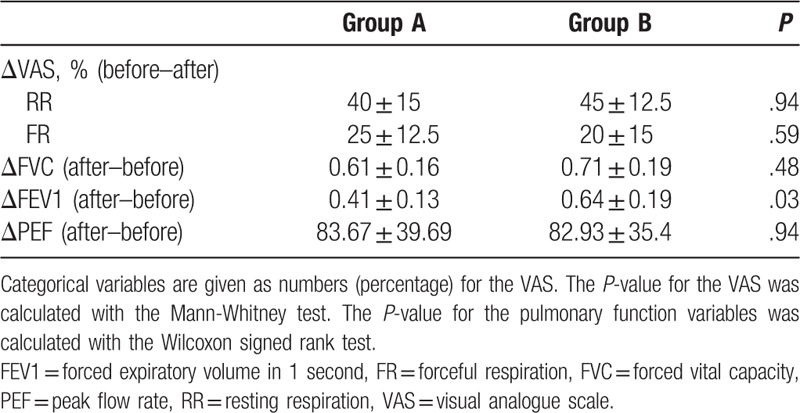
Comparison of the equivalence of the effectiveness of treatment between both groups.

### 3.5 Other factors related to the effectiveness of the intervention

There was a statistically significant impact of the number of fractures on ΔVAS during forceful respiration (coefficient = 0.536, *P* = .04) (Table 4). However, the variables in the PF test did not significantly impact the number of fractures on ΔVAS during forceful respiration (ΔFVC, *P* = .19; ΔFEV1, *P* = .21; ΔPEF, *P* = .30). The age of patients was shown to have a statistically significant influence on ΔPEF (coefficient = −0.629, *P* = .002).

**Table 4:**
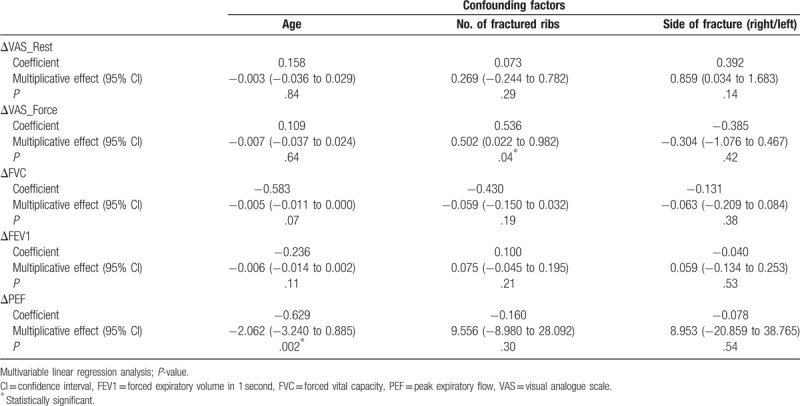
Confounding factors related to the effectiveness of intervention.

## 4 Discussion

In this study, we compared 2 types of splints for RF patients. One was a manufactured product, and the other was constructed in the ER using dressing material, double-sided tape, and a splint.

Pain control is the core goal of treatment for patients with RFs.^[4,5]^ Numerous methods for treatment of RFs have been suggested; however, none of these methods has been shown to be completely effective alone.^[5]^ Therefore, for maximal effectiveness, several methods must be combined.^[5]^ However, these methods expose patients to risks of side effects or iatrogenic complications.^[5]^

For these reasons, the rib splint was designed as an alternative method to the treatment of patients with RFs. Initially, application of a rib splint reduced pain by stabilizing the fracture site. However, because the initial rib splints decreased the vital capacity of the lung^[7]^ they were not widely used. At present, rib splints that can simultaneously increase vital capacity and reduce pain have been developed and manufactured.^[7,8]^ However, this product is very expensive, and its size is inadequate for application to patients with multiple RFs or a flail chest. Therefore, we have designed a rib splint that was simply constructed using common materials available in the ER, such as adhesive hydrocolloid dressing material, double-sided adhesive tape, and a splint. Then, we applied this form of rib splint to patients with RFs.

We have investigated the difference in the VAS score and PF variables between before and after intervention, in which the CCO rib splint or the ER splint was applied, during resting and forceful respiration.

Based on the VAS and PF variables, no statistically significant difference was detected between applying the CCO rib splint and the ER splint. Furthermore, the CCO rib splint and the ER splint statistically significantly reduced the VAS score after rib splint application compared to before the intervention.

Several confounding factors have been identified. The number of fractured ribs and age significantly affect the ΔVAS during forceful inspiration and ΔPEF, respectively. The ΔVAS during forceful inspiration increased by approximately 0.5 as the number of fractures increased by one. The initial pain scale in patients with more RFs is higher than those with fewer fractures. Assuming that the pain experienced by both groups of patients was effectively reduced after applying ER rib splint such that they feel comfortable, the pain scale after rib splinting in both groups are likely to be similar. As a result, the subjective pain reduction (ΔVAS) may be increased when the number of fractures increases. In other words, this result suggests that the ER splint is more effective in patients with more RFs in reducing pain, even though the maximum number of fractures was 4 in this study. The ΔPEF was decreased by approximately 0.6 as the age was increased by one. The PEF is likely to decrease with age. Thus, the extent of improvement in PEF with the splint will decrease as the age increased. Pain reduction is the most important consideration for RF patients because pulmonary complications due to hypoventilation resulting from pleuritic pain exert an important influence on the prognosis of these patients.^[2–5]^ Therefore, several studies have examined pain control in RF patients. Regarding invasive treatment methods, Hashemzadeh et al^[9]^ compared the use of thoracic epidural blockers with the use of intercostal blockers. They reported that thoracic epidural blockade was superior to intercostal blockade in providing pain relief from RFs.^[9]^ In Korea, Hwang and Lee^[10]^ reported the effectiveness of intercostal nerve blockade for patients with RFs. However, according to the results reported by Kieninger et al,^[11]^ epidural blockade is associated with prolonged length of stay and increased complications in elderly patients with RFs, particularly those with less significant injuries, regardless of cardiopulmonary comorbidities.

Concerning more invasive methods, Nirula et al^[12]^ reported in 2006 that RF fixation may reduce ventilator requirements in patients with severe thoracic injuries but that the long-term functional outcomes of such a surgical procedure need to be assessed. In 2010, Nirula Mayberry^[13]^ reported that surgical fixation methods may be effective in decreasing RF pain but may also increase the risk of iatrogenic bleeding or pulmonary damage. Because surgical fixation methods are invasive, iatrogenic complications are possible. Therefore, the safety of these methods should be considered when treating RF patients.

Several studies have examined less invasive or noninvasive treatment methods. For instance, Solak and Öz ^[14]^ investigated the effectiveness of transdermal opioids for patients with RFs and reported no significant difference in effectiveness between transdermal opioid combined with intercostal nerve blockade and IV patient-controlled analgesia combined with intercostal nerve blockade. They concluded that transdermal opioid treatment is safe, noninvasive and effective for patients with RFs.^[14]^ Similarly, Zink et al reported no significant difference in effectiveness between a lidocaine patch and narcotic administration. Thus, they concluded that the lidocaine patch is a safe, effective treatment method.^[15]^

However, Ingalls et al^[16]^ found no significant difference in effectiveness between a lidocaine patch and a placebo patch; thus, they concluded that the lidocaine patch does not significantly reduce pain in patients experiencing multiple traumas with RFs. In Japan, Nakae et al^[17]^ reported that the difference in the effects on RF-related pain between the traditional Japanese medicine *Jidabokuippo* and NSAIDs was not significant. Bayouth et al^[18]^ reported that early IV administration of ibuprofen reduced the requirement for narcotics and the length of stay among patients with RFs.

No previous study had compared the effectiveness between the CCO rib splint and a simply constructed rib splint made in the ER. Based on the results of this study, this less expensive rib splint that was simply constructed in the ER expands the applicability of a rib splint for patients with RFs and significantly reduces RF-related pain.

Nevertheless, there were several limitations in this study. First, there was selection bias because we enrolled only patients lacking damage to internal organs in the thoracic cage. The effectiveness of rib splints for patients with RFs could be exaggerated because this study did not enroll patients with traumatic pneumothorax, hemothorax, lung contusion, etc. Another limitation of this study is that we excluded multiple trauma patients, who experience injuries to sites in addition to the thorax. However, based on the results of this study, we suggest that the less expensive ER splint can be effective in patients with only RFs.

Second, we used minimum sample size. However, we have calculated the sample size based on the results of pilot study. It is possible that the ER splint provides similar benefits to those of the CCO rib splint. Therefore, based on the findings of this study, we will perform further evaluations of length of stay, requirement for analgesics and narcotics, etc.

Third, some results of this study were not quantitative, and the VAS score is insufficient to establish the effectiveness of the intervention. However, we have evaluated the PF of patients before and after interventions.

Fourth, we did not follow up the enrolled patients. As a result, we did not analyze the patients after they left the ER. Accordingly, we did not compare the effectiveness between the CCO rib splint and the ER splint over time.

Fifthly, we did not verify the durability of the ER splint because we did not follow up the enrolled patients. However, judging by the results of this study, if the durability of the ER splint is similar to that of the CCO, we hypothesize that the results of follow would be similar as well.

## 5 Conclusion

In conclusion, both the CCO rib splint and the less expensive ER splint effectively reduced pain and improved PF in patients with RFs.

## Author contributions

**Conceptualization:** YOONJE LEE, Sang-Hyun Lee, Changsun Kim, Hyuk Joong Choi.

**Data curation:** Hyuk Joong Choi.

**Formal analysis:** YOONJE LEE, Sang-Hyun Lee.

**Investigation:** YOONJE LEE, Sang-Hyun Lee.

**Methodology:** YOONJE LEE, Sang-Hyun Lee.

**Supervision:** Changsun Kim.

**Validation:** Changsun Kim.

**Writing – original draft:** YOONJE LEE.

**Writing – review & editing:** Sang-Hyun Lee.
